# Wearable Sensors for Health Monitoring

**DOI:** 10.3390/bios16020093

**Published:** 2026-02-02

**Authors:** Caroline Abreu, Carla Bédard, Jean-Christophe Lourme, Benoit Piro

**Affiliations:** 1 ITODYS, CNRS, Université Paris Cité, 75006 Paris, France; 2 ValoTec, 1 Mail du Professeur Georges Mathé, 94800 Villejuif, France

**Keywords:** health monitoring, wearable sensors, implantable sensors, biomarkers, sensors, biochemical monitoring, body fluid analysis, screening and early diagnostics

## Abstract

The growing global population and the rapid increase in older adults are driving healthcare costs upward. In response, the healthcare system is shifting toward models that enable continuous monitoring of individuals without requiring hospital admission. Advances in sensing technologies, embedded systems, wireless communication, nanotechnology, and device miniaturization have made these smart systems possible. Wearable sensors can monitor physiological indicators and other symptoms, helping to detect unusual or unexpected events. This allows for the provision of timely assistance when it is needed most. This paper outlines the challenges associated with these systems and reviews recent developments in wearable, sensor-based human activity monitoring. The focus is on health monitoring applications, including relevant biomarkers, wearable and implantable sensors, and established sensor technologies currently used in healthcare, as well as future prospects. It also discusses the challenges involved in researching, developing, and applying these sensors. The goal is to promote the widespread use of these sensors in human health monitoring.

## 1. Introduction

The rapid evolution of sensing technologies, flexible electronics, and wireless communication over the past several decades has transformed the way physiological signals can be measured and interpreted. Among these advancements, wearable sensors have emerged as powerful tools capable of continuously monitoring key health indicators in real time. Their growing popularity in security, entertainment, commercial, and especially medical fields reflects a broader shift toward decentralized, personalized, and preventive healthcare models. As global populations age and chronic diseases become more prevalent, the demand for systems that enable unobtrusive, long-term monitoring outside clinical settings continues to grow.

In the medical field, wearable and implantable sensors provide a unique opportunity to assess human activity and physiological function with high temporal resolution. These devices can non-invasively measure vital parameters, including cardiovascular signals, body temperature, biochemical markers, and metabolic indicators, providing insights that were previously accessible only through hospital-based equipment. Wearable sensors capture dynamic physiological changes, helping to detect early signs of illness, monitor disease progression, and support timely interventions. This improves quality of life and reduces healthcare costs.

Recent advancements in materials science, nanotechnology, and printed electronics have enabled the creation of highly flexible, stretchable, and biocompatible sensing platforms that conform closely to the human body. These technologies can now measure complex biochemical analytes in sweat or interstitial fluid, track motion and posture, and continuously monitor heart rate, blood pressure, and temperature. Progress in miniature power systems and wireless data transmission supports the seamless integration of these technologies into everyday life.

This review provides an overview of the current state of wearable and implantable sensors for health monitoring. It emphasizes key physiological signals and biomarkers, describes major sensing mechanisms and materials, and explores recent advancements in device design and fabrication. Additionally, it outlines the current challenges and prospects for translating these technologies into robust, user-friendly systems suitable for widespread adoption in healthcare.

**Figure 1:**
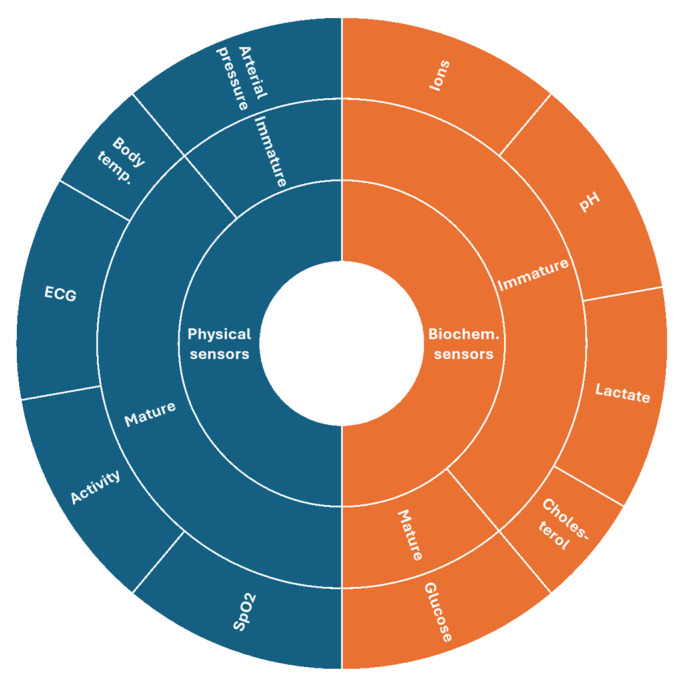
This schematic diagram summarizes the various applications of wearable sensors reviewed in this article, depending on their type (physical or chemical) and maturity level.

The cited works were selected based on their significance and adequacy to the message we chose to deliver. There were no fixed inclusion or exclusion criteria. Google Scholar was used as the search engine, and no precise time window was used. Significant review articles are systematically cited at the end of each section so that readers can access various illustrated examples. Figure 1 summarizes the various applications of physical and biochemical sensors reviewed in this work and their maturity levels.

This manuscript provides an integrated analysis of biochemical, physiological, and implantable biosensing technologies, linking printing-based fabrication strategies with technological maturity. It distinguishes clinically validated systems (e.g., CGM, ECG, and SpO_2_) from emerging wearable biochemical sensors that target sweat and interstitial fluid. By contextualizing laboratory-scale innovations within real healthcare pathways, this review provides a realistic view of translational readiness. This work is further distinguished by the inclusion of textile-based, tattoo-based, and fully printed biosensing platforms, providing a forward-looking resource for the wearable biosensor community.

## 2. Wearable and Implantable Health Monitoring Sensors

### 2.1. Wearable Sensors

Wearable sensors are the primary tools for detecting physiological signals from the human body. These devices can measure various parameters, such as blood glucose levels, heart rate, respiratory rate, and body motion [1]. Each parameter provides essential information about health and bodily function, and the underlying mechanisms through which the sensors detect them differ.

#### 2.1.1. Physiological Sensors

##### Cardiovascular Monitoring System

Continuous monitoring of heart rate and pulse is essential for the prevention and diagnosis of cardiovascular diseases (CVDs). The heart’s primary function is to pump blood throughout the body, thereby maintaining tissue perfusion, recycling venous blood, and enabling metabolic exchange processes [2]. Heart rate reflects the frequency of cardiac cycles, and in clinical settings, heart rate and pulse are fundamental indicators of cardiac function [3,4].

Cardiovascular disease (CVD) remains one of the leading causes of death worldwide, responsible for more than 17 million deaths annually—approximately 31% of all deaths globally [5]. This number is projected to rise to 23.6 million by 2030 [6]. Despite the high mortality rate, up to 90% of cases of cardiovascular disease (CVD) are preventable through early detection [7]. Heart rate and pulse provide vital information that supports the timely prevention and treatment of cardiovascular conditions [8]. Although cardiovascular diseases (CVDs) are typically asymptomatic in their early stages, they affect arterial pulse characteristics, influence blood pressure, and alter wrist pulse waveforms [9].

Traditional cuff-based sphygmomanometers are still widely used in clinical practice to measure systolic and diastolic blood pressure [10,11]. However, this indirect method does not enable continuous monitoring. To overcome this limitation, the authors proposed a flexible blood pressure sensor based on ultrathin, lightweight graphene tattoo sheets [12]. When placed over a wrist artery, a small electrical current is applied to the skin to measure bioimpedance. Machine learning models then correlate fluctuations in this signal with blood pressure, enabling continuous monitoring. Another study introduced a flexible blood pressure sensor based on piezoelectric composite ultrasonic technology, which represents a significant departure from conventional approaches [13]. In this design, polydimethylsiloxane (PDMS) and conductive silver nanowires were integrated using a dice-and-fill technique, yielding a highly flexible sensor.

The electrical activity of the heart is commonly assessed using an electrocardiogram (ECG), which provides essential insights into cardiovascular health. Because ECG signals are periodic, heart rate can be derived from R-to-R (RR) intervals [2]. Traditional ECG monitoring relies on gel-assisted Ag/AgCl electrodes, but these can be inconvenient [14]. In contrast, skin-contact ECG sensors allow users to more easily monitor cardiac health and detect early signs of serious conditions, such as cardiomyopathy, arrhythmias, and hypertension [15,16]. However, Ag/AgCl electrodes may cause skin irritation [14,17], which has driven increasing interest in flexible dry electrodes. One example is a flexible, wearable electrode in which highly conductive silver was deposited onto cowhide via plasma sputtering [18]. Tests on six subjects showed that the resulting ECG signal quality was comparable to that obtained using standard Ag/AgCl electrodes.

Plethysmography and ultrasound are widely used to monitor pulse and heart rate [19], but the bulky equipment and difficulty maintaining accuracy during long-term measurements limit their use in wearable systems [20,21]. To address these limitations, a flexible strain sensor for real-time pulse monitoring was recently introduced [22]. This patch-type device uses highly sensitive, flexible polyaniline to detect pressure changes caused by blood flow. This allows for the analysis of pulse characteristics for clinical applications. Further developments have led to the creation of a flexible pressure sensor capable of monitoring three pulse positions simultaneously [23]. This device integrates traditional Chinese pulse diagnosis with modern sensor technology. Ionogel-based pressure sensor arrays were fabricated on PET substrates, converting arterial pressure fluctuations into changes in electrical resistance. One advantage of this system is its ability to generate three-dimensional pulse maps that closely mimic a physician’s tactile assessment by presenting key information, including pulse strength and waveform.

**Figure 2:**
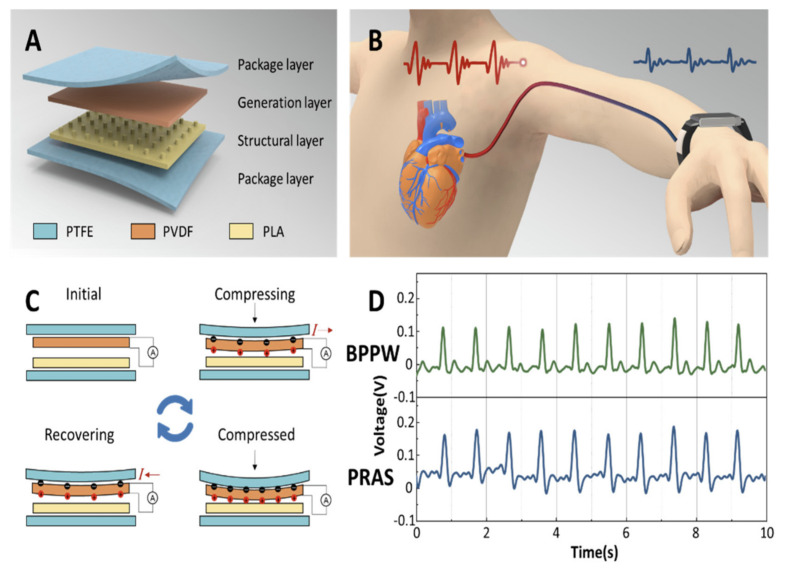
(**A**) Structure and materials used in the pressure sensor. (**B**) Artistic view of the working principle. (**C**) Detail of the piezoelectric transduction: bending generates charges, i.e., a voltage difference, collected into the external measurement circuit. (**D**) Comparison between the signal from this blood pressure prediction wristband (BPPW) and a commercial physiological recording analyzer system (PRAS). Reproduced from Tan et al. [25]. Open access figure distributed under the terms and conditions of the Creative Commons Attribution (CC BY) license.

Another successful example of a flexible pulse sensor is based on poly (vinylidene fluoride-co-trifluoroethylene) (PVDF-TrFE) [24]. This device integrates analog amplification circuitry and sensing components onto a flexible substrate that can detect low pressures (down to 10 kPa) and increase electrical signal strength tenfold. These characteristics make it highly effective for monitoring pulse signals in humans. Figure 2 shows a wearable device that monitors arterial pressure through the piezoelectric effect.

The reader can refer to the review article from Prieto-Avalos et al. [26] for a good overview of the literature on cardiovascular monitoring systems. It should be noted that none of the cuffless blood pressure devices reviewed above have reached a high level of maturity yet. For this reason, publications rarely present stringent validation protocols and never refer to standards, particularly those of the AAMI/ESH/ISO (Association for the Advancement of Medical Instrumentation/European Society of Hypertension/International Organization for Standardization). One may conjecture that standards and protocols will become more prevalent for more mature systems in the near future.

##### Activity Monitoring System

The monitoring of motion or activity of moving objects is a widely studied topic in the field of wearable electronics. Most wearable motion-detection systems rely on strain sensors, which use variations in baseline resistance to indicate motion-related activities [27,28]. Motion detection is particularly valuable for prosthetic limbs and soft robotics, as well as for individuals with physical impairments and elderly persons who require continuous remote activity monitoring [29,30]. However, many state-of-the-art sensors and systems have significant drawbacks, including bulkiness, rigidity, limited wearability, and excessive weight, which make continuous monitoring difficult [29,30]. Consequently, substantial research efforts have focused on developing lightweight, wearable systems that use thin-film electronics and sensors printed directly onto flexible polymeric or textile substrates [31]. These wearable platforms, often referred to as electronic skins, are equipped with various sensors designed to mimic the sensory functions of human skin [32,33]. Various strategies have been explored to create these sensors, including using discrete sensing elements distributed across multiple body regions or fully integrated systems embedded within connected wearable suits [30,34]. Soft motion-sensing suits typically incorporate sensors positioned at joints that articulate during movement, thereby activating the sensing elements. One application is monitoring human gait, where strain sensors embedded in a wearable suit capture motion patterns [35]. In another development, strain sensors made from liquid metal and embedded in elastomers were placed on the hip, knee, and ankle joints to measure bending angles [29].

Nanoscale materials play a significant role in the design of motion-detection sensors, whether in their pure form or mixed with elastomeric polymers as composites. Embedding conductive fillers into rubber-based matrices is advantageous because of the matrices’ high stretchability and ease of integration onto non-planar surfaces. For example, natural rubber infused with liquid-exfoliated graphene can form a conductive composite [36]. This composite exhibited a 10^4^-fold increase in resistance at strains up to 800%, demonstrating high sensitivity and a dynamic response suitable for continuous motion monitoring when mounted on joints or other moving body parts. Silver nanowires printed with an Ecoplex dielectric layer have also been used to fabricate capacitive sensors for multifunctional wearable applications [37]. These sensors can detect strain, pressure, temperature, and touch under various physiological conditions.

Another notable approach used a double-helical carbon nanotube (CNT) array to create a strain sensor for hand motion detection [38]. This design allowed for strain measurements of up to 410% with low hysteresis and high sensitivity to subtle movements. Additionally, CNT/Ecoflex nanocomposites have been used to produce ultra-stretchable, skin-mountable strain sensors for motion monitoring [39]. In these composites, the CNT percolation network forms conductive pathways while the Ecoflex matrix accommodates large deformations associated with joint or muscle motion. This results in resistance changes that correspond to expansion levels.

Further work has explored the 3D printing of CNT/polyurethane nanocomposites, in which thin extruded filaments serve as strain sensors [40]. Similarly, CNT/PDMS nanocomposites have shown great promise for wearable, strain-based motion detection [41,42,43]. 3D printing enables precise control over CNT/polyurethane mixing ratios and viscosities, facilitating the fabrication of composites optimized for percolation and ideal for strain-sensing applications. In another development, a multifunctional sensing device was printed on a flexible substrate that incorporated Ag, CNTs, PEDOT:PSS, and ZnO to detect multiple physiological signals in real time [44]. These sensor patches comprise disposable and non-disposable components. The disposable component adheres to the skin and integrates printed sensors that track temperature, acceleration, and ECG signals. It also includes kirigami structures that accommodate skin deformation.

Recent advances include the development of a novel composite consisting of a conductive sponge made of carbon black impregnated in a shear-thickening gel and polyurethane [27]. This sensor provides effective impact protection, reducing applied forces by 44%, while simultaneously monitoring human motion. Additionally, strain sensors based on liquid metals and conductive ionoelastomers have garnered considerable interest due to their ability to detect large deformations with high sensitivity [45,46].

Chang et al. [47] provided an overview of the literature on activity monitoring systems.

##### Body Temperature Monitoring System

Wearable temperature sensors have been widely studied, with significant exploration of various sensing materials and printing technologies. In wearable applications, these sensors serve two primary purposes: continuous monitoring of body temperature and ambient temperature measurement. For body temperature monitoring, sensors are typically mounted directly on the epidermis or maintained in close contact with the skin as detachable components of wearable devices [48]. Continuous body temperature tracking is important for patients with chronic illnesses, unconscious or injured individuals, people undergoing anesthesia or surgical procedures, and workers exposed to extreme environmental conditions. Wearable temperature sensors are also crucial in electronic skin technologies and are frequently investigated alongside other sensing systems aimed at advancing industrial and social robotics [49,50].

Printed thermal sensors typically operate by measuring changes in the resistance of metallic structures as the temperature increases. Thermal coefficient of resistance (TCR) values are used to quantify their response [51]. In recent years, numerous thermal sensors have been developed using intrinsically conductive materials and nanocomposites engineered into flexible, minimally constrained geometries [52,53].

Fully printed fabrication methods simplify manufacturing, reduce costs, and enable the creation of single-layer, patterned, conductive lines of various geometries, such as meanders, spirals, and circles. For instance, a polymeric blend containing SWCNTs was used to detect both temperature and CO_2_ gas simultaneously [54]. Direct attachment of sensors to the epidermis requires substrates with skin-like properties, such as biocompatibility, breathability, oxygen permeability, and waterproofing. Meeting all these requirements remains challenging, however. To address this challenge, breathable and stretchable temperature sensors inspired by human skin have been developed [55]. Additional work includes transfer-printed copper strips on semipermeable polyurethane films and an inkjet-printed graphene/PEDOT:PSS temperature sensor fabricated on a skin-conformable polyurethane substrate, which eliminates the need for photolithography [56].

Skin-mounted biosensors that can detect sweat metabolites, electrolytes, and temperature simultaneously have been integrated into a single, wearable patch designed for prolonged use during exercise [57]. A related approach enabled the concurrent measurement of sweat pH using an ion-selective field-effect transistor (ISFET) and skin temperature using an embedded temperature sensor [58]. Silver-based interconnects were printed, and poly (3,4-ethylenedioxythiophene):poly (styrene sulfonate) (PEDOT:PSS) served as the temperature-sensing material. Real-time testing was performed by attaching the patch to a subject’s neck while they exercised.

Recent advances have investigated the printing and performance of nanoscale sensing materials. The resistance variations of sensing layers connected by conductive pastes were evaluated under temperature changes. A comparative study of carbonaceous materials, including reduced graphene oxide (rGO), single-walled carbon nanotubes (SWCNTs), and multi-walled carbon nanotubes (MWCNTs), assessed responsivity and long-term stability [59]. Reduced graphene oxide (rGO) exhibited strong, stable performance under varying environmental conditions, including humidity, pressure, and gas exposure. It remained responsive even when coated with protective insulating layers. Graphene nanowells exhibited exceptionally high TCR values (up to 180% K^−1^), making them promising candidates for monitoring human body temperature [60].

A biocompatible, conductive, “green” electrolyte composed of aliphatic diol–calcium chloride (CaCl_2_) complexes was identified as a promising material for in vivo and in vitro temperature sensing. It showed consistent resistance changes with temperature [53]. A customizable, 3D-printed sensor capable of monitoring temperature and pressure was developed using a skin-toned substrate for direct visual readout [61]. Through multilayer 3D integration, pressure and temperature sensors were combined into a single, flexible patch. Furthermore, an innovative, self-adhesive mechanism inspired by octopus suction cups was created using PDMS microstructures to ensure robust adhesion to the skin [62].

A resistance-based temperature sensor was developed using a nanocomposite of poly (N-isopropylacrylamide) (PNIPAM) hydrogel, poly (3,4-ethylenedioxythiophene):poly (styrene sulfonate) (PEDOT: PSS), and carbon nanotubes (CNTs). This sensor exhibits a strong thermal response of 2.6% °C^−1^ within the human temperature range (25–40 °C). A highly stretchable, self-healing thermistor based on a polyacrylamide/carrageenan double-network hydrogel demonstrated stretchability of up to 330% strain and sensitivity of 2.6% °C^−1^ at high strain levels [63], making it suitable for placement on joints or irregular surfaces. This thermistor closely mimics the self-healing behavior of human skin.

Precise temperature feedback is essential for monitoring active heating in applications such as heat therapy, perioperative care, and controlled transdermal drug delivery. Integrated wearable temperature sensors have been used for these purposes [64]. Stretchable aluminum heaters combined with gold-based resistance temperature detectors (RTDs) were implemented in a tattoo-like wearable patch. This patch was fabricated using a cut-and-paste method and can be attached to any body area without restricting movement [63]. These developments collectively highlight the strong and growing interest in advancing wearable temperature-sensing technologies across diverse application domains.

**Figure 3:**
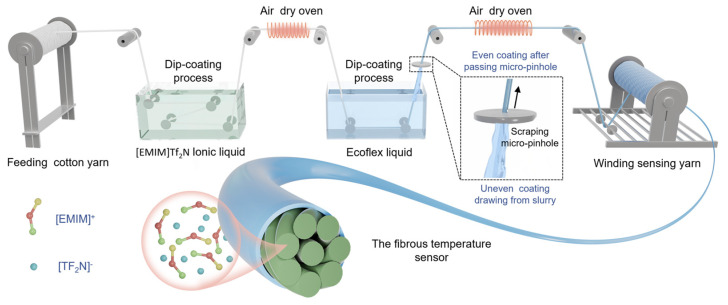
Schematic illustration of the fabrication of a fibrous temperature sensor. Reproduced from Li et al. [65]. Open access figure distributed under the terms and conditions of the Creative Commons Attribution (CC BY) license.

Due to their flexibility, comfort, and compatibility with textile integration, fiber-based temperature sensors are of increasing interest for wearable physiological monitoring. Li et al. [65] described a temperature-sensitive fiber containing an ionic liquid that demonstrates stable and repeatable thermal responses with good linearity and high sensitivity (2.6% °C^−1^; Figure 3). The sensor’s performance remains largely unaffected by mechanical stresses, such as compression, bending, and twisting. Stable operation is also maintained under acidic and alkaline conditions. Furthermore, this fiber sensor has been successfully incorporated into firefighter protective garments, sports wristbands, and infant clothing, enabling continuous body temperature monitoring and early-warning functionality.

As described above, such temperature sensors only target skin temperature. It is usually lower and much more variable (it can vary from 28 to 34 °C) than blood temperature (36.2–37.5 °C for healthy individuals) depending on environmental factors, clothing, blood flow, and body area. Therefore, skin temperature does not directly reflect core temperature and the transfer function is only valid for some given areas (armpit, ear).

For an overview of the literature on wearable body temperature sensors, readers can refer to Hashimoto et al.’s review article [66].

##### Blood Oxygen Saturation (SpO_2_) Monitoring System

Blood oxygen saturation (SpO_2_) is a vital sign that reflects the percentage of oxygen-bound hemoglobin in the blood. Continuous SpO_2_ monitoring is essential for managing respiratory diseases, cardiovascular conditions, sleep disorders, and critical care [67]. Wearable SpO_2_ technologies integrated into smartwatches, rings, earbuds, and skin patches have rapidly evolved from traditional clinical pulse oximeters into compact, low-power platforms capable of long-term, noninvasive physiological tracking. Advances in optical sensing, noise-reduction algorithms, flexible electronics, and wireless connectivity have driven this evolution.

These wearable systems enable real-world physiological assessments and offer trend-based insights into conditions such as sleep apnea, chronic obstructive pulmonary disease (COPD), and silent hypoxemia. During the pandemic, wearable oximeters became widely used for remote patient monitoring, highlighting their clinical relevance [67]. Beyond conventional fingertip devices, modern wearable oximeters provide continuous, unobtrusive monitoring of blood oxygenation in various forms.

Despite significant progress, there are still accuracy challenges, particularly under high-motion conditions, with individuals of darker skin tones, and in low-perfusion states. Improving calibration strategies, ensuring equitable performance, and lowering energy consumption remain important goals for future development. As these technologies continue to advance, wearable SpO_2_ systems are poised to play an increasingly important role in telemedicine, chronic disease management, sleep diagnostics, and personalized health monitoring.

Dcosta et al. [68] provided an overview of the literature on SpO_2_ monitoring systems.

#### 2.1.2. Biological Fluid-Based Sensors

##### Glucose Sensors

Diabetes is a widespread chronic disease that poses serious global health risks. According to the World Health Organization (WHO) [69,70], millions of individuals are affected worldwide, and diabetes remains one of the leading causes of mortality each year. The primary cause of diabetes is dysfunctional or depleted insulin production, which leads to irregular glucose levels. Therefore, frequent glucose concentration monitoring is essential to prevent severe complications. Since the introduction of the first-generation glucose biosensor by Clark and Lyons [71] at Cincinnati Children’s Hospital in 1962, extensive development of these devices has occurred. Various human physiological fluids, including blood, urine, sweat, saliva, interstitial fluid, ocular fluid, and breath, contain glucose biomarkers suitable for diagnostic purposes [72,73].

Enzymatic sensing, particularly the use of glucose oxidase (GOx), remains the most widely adopted method for selective glucose detection [73]. In this approach, redox reactions at the sensor interface generate a measurable decrease in oxygen concentration and release hydrogen peroxide (H_2_O_2_), both of which are proportional to glucose levels. Initially developed for laboratory blood analysis, glucose monitoring technologies have rapidly evolved into handheld devices for rapid point-of-care testing. Modern glucose meters typically rely on disposable, enzyme-activated electrode strips [73]. Comprehensive reviews of glucose sensors and recent advancements in portable detection systems can be found elsewhere [72,73,74,75].

Due to the rapid growth of microelectronics on unconventional substrates, especially wearable and conformable materials, glucose sensors have attracted substantial research interest [76]. Wearable and implantable glucose sensors enable in vivo measurements, allowing for continuous, real-time glucose tracking and improved self-management of diabetes [77].

Emerging technologies, such as the Internet of Things (IoT), enable clinicians to remotely monitor patient data through cloud-based systems [78]. Several recent proof-of-concept devices have demonstrated practical, real-time glucose monitoring capabilities. One example is an all-printed, tattoo-based glucose sensor designed for noninvasive glycemic monitoring [79]. This device operates through epidermal sensing, combining reverse iontophoretic extraction of interstitial glucose with enzyme-based amperometric detection. Screen printing was used to fabricate the electrodes: a Papilio transfer-paper substrate with silver (Ag) and silver chloride (AgCl) as the reference and counter electrodes, respectively, and conductive carbon ink as the working electrode. Comparative in vitro and on-body measurements demonstrated the device’s strong potential for detecting micromolar glucose concentrations.

Another innovative development is a wearable sensor array for multiplexed perspiration analysis [80]. This fully integrated platform can simultaneously monitor multiple sweat biomarkers, including glucose, lactate, sodium, and potassium ions, as well as skin temperature. The electrochemical glucose and lactate sensors use Ag/AgCl electrodes, and ion-selective membranes are drop-cast for electrolyte detection. Tests performed during indoor and outdoor physical activities confirmed the system’s effectiveness for personalized diagnostics and real-time health monitoring.

Functional nanoscale materials, including nanowires, nanoribbons, and nanotubes, are important for designing conformable, wearable sensors. For example, indium oxide (In_2_O_3_) nanoribbons were used to fabricate field-effect transistor (FET) sensors on polydimethylsiloxane (PDMS) substrates [81]. After being laminated onto the skin, these sensors successfully detected glucose in sweat, tears, and saliva at concentrations as low as 10 nM. Another system integrated a sweat-based glucose sensor with a closed-loop transdermal drug delivery module, enabling accurate glucose measurement with real-time correction for pH, temperature, and humidity variations [82]. These miniaturized devices were fabricated via clean-room processes and transferred onto PDMS for skin-conformable operation.

Other wearable sensing platforms include cotton fabrics printed with carbon, graphite, silver (Ag), and silver chloride (AgCl) electrodes for lactate detection [83]. In another example, a spray-printed reduced graphene oxide (rGO) working electrode was integrated into a wrist-mounted sensor, yielding excellent amperometric responses for in vivo glucose detection in the 0–2.4 mM range [84]. A robust, non-enzymatic, wearable sensor patch incorporating integrated signal processing and wireless communication modules was developed on flexible stainless steel [77]. Implanted into subcutaneous tissue, these sensors continuously monitor interstitial glucose levels.

Similarly, organic-material-based biosensors have shown promise due to their compatibility with low-temperature fabrication processes and their inherent biocompatibility. One such device uses a cross-linked film of PEDOT (poly (3,4-ethylenedioxythiophene)) and glucose oxidase for amperometric glucose sensing [78]. A three-electrode system patterned on a PET substrate was paired with front-end electronics to enable remote glucose monitoring.

Together, advances in sensing materials, device architectures, and fabrication methods demonstrate the rapid progress of this field. Minimally invasive, wearable glucose-monitoring technologies have been industrialized for over 20 years. Non-invasive sensors, which are not yet fully mature, will likely appear on the market in the near future.

To accelerate development, existing commercial devices can be adapted. This allows you to use robust and reliable components while only developing the specific sensing part. Chen et al. [85] recently achieved this by adding a “glucose add-on” to a commercial smartwatch bracelet. Consequently, the watch can provide information on mapping, physical activity, heart rate, and glucose levels. This helps develop the glucose sensor and identify trends in glucose levels (e.g., an unexpected drop in glucose levels when inactive versus an expected drop during heavy activity). In this example, the glucose sensor is powered by the smartwatch and communicates directly with it.

Glucose monitoring systems have been reviewed by Xue et al. [86], for example.

##### Lactate Sensors

Lactate, a key metabolite produced in muscle tissue during anaerobic glucose metabolism, plays a crucial role in the human body. Continuous lactate monitoring is important during physical activity, especially for athletes, to prevent cell acidosis, which can severely impair muscle performance [87,88]. Several human body fluids contain measurable lactate concentrations. For instance, the lactate concentration in blood is typically 0.5–1.5 mM in healthy individuals at rest, rising to approximately 12 mM during intense exercise [89]. Other fluids, such as tears, saliva, and sweat, exhibit distinct lactate concentrations [87,89,90]. For practical, wearable applications, lactate sensors should be noninvasive and operate using sweat or interstitial fluid analysis [91,92].

Selectivity is essential because body fluids contain numerous other metabolites. Most wearable electrochemical lactate sensors use enzymes, such as lactate oxidase, for selective detection. However, non-enzymatic approaches have also been explored [93]. Redox mediators are sometimes incorporated to enhance catalytic activity and improve sensing performance.

An electrochemical biosensor in the form of a printable tattoo was developed for real-time lactate monitoring during exercise [88]. This device uses Ag/AgCl electrodes and functionalized multi-walled carbon nanotubes (MWCNTs) in a three-electrode configuration. The biosensor exhibited strong chemical selectivity for lactate, demonstrated linear detection up to 20 mM, and maintained stable performance under continuous mechanical deformation. A multiplexed sensor array capable of measuring multiple metabolites, electrolytes, and skin temperature simultaneously was also demonstrated on a soft, flexible substrate [94]. This fully integrated, wearable patch can be comfortably worn on the wrist or as a headband.

Hybrid sensing systems that combine biochemical and electrophysiological monitoring represent an advanced approach to wearable health tracking [95]. In one such system, Ag/AgCl electrodes were screen-printed, and lactate oxidase was used as the selective enzyme. When mounted on the skin, the patch enabled the simultaneous measurement of electrical and chemical signals without cross-interference. A non-enzymatic lactate sensor was fabricated using screen printing. In this sensor, the working electrode was electropolymerized with 3-aminophenylboronic acid (3-APBA) that was imprinted with lactate [93]. This device achieved a detection range of 3–100 mM, a detection limit of 1.5 mM, and a response time of two to three minutes.

Textile-based, printed amperometric biosensors have also been demonstrated for lactate monitoring [96]. Carbon, graphite, and Ag/AgCl inks were deposited onto cotton fabric for the working, reference, and counter electrodes, respectively. After lactate oxidase immobilization, the biosensors achieved a detection range of 0.05–1.5 mM within five minutes. A tube-shaped painted biosensor also demonstrated strong potential as a portable, user-friendly biochemical sensing platform [92]. Its interior surface was printed with carbon graphite and Ag/AgCl to form an electrochemical cell suitable for lactate detection.

Furthermore, graphene nanowells were printed alongside Ag/AgCl electrodes to create an electrochemical biosensor capable of real-time lactate detection [97]. Testing in fluids such as deionized water and phosphate-buffered saline revealed a broad detection range of 1.0 μM to 10 mM, which can mimic various physiological environments.

**Figure 4:**
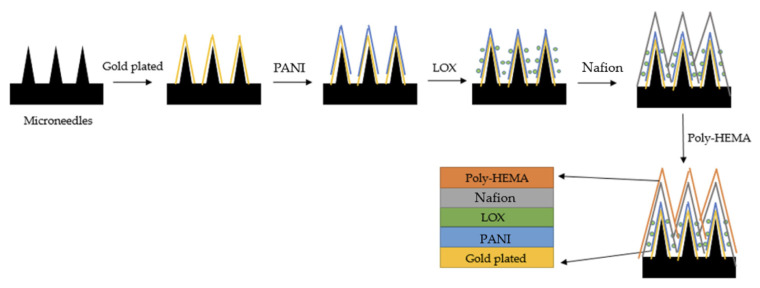
Schematic of an enzymatic percutaneous microneedle array for lactate sensing. Reproduced from Chien et al. [98]. Open access figure distributed under the terms and conditions of the Creative Commons Attribution (CC BY) license.

Similar to continuous glucose monitoring, continuous lactate sensing is being investigated using percutaneous microneedle arrays. For example, Chien et al. [98] coated the base of a percutaneous microneedle array with an electroplated conductive polymer layer of polyaniline (PANI). Then, a lactate oxidase (LOx) layer was deposited. Then, a Nafion-modified polymer layer was applied to enhance the selective adsorption of the target analyte at the electrode surface. Finally, a protective poly (HEMA) film was introduced as the outermost layer to isolate the electrode from interfering impurities. Figure 4 provides a schematic illustration of the multilayer structure. Each needle is 1 mm long, 0.44 mm wide, and 0.03 mm thick. This device has a lactate detection range of 0.5–40 mmol L^−1^.

Lactate sensing remains a major focus of research in the field of wearable electronics. The rapid progress in sensing materials, device integration, and fabrication techniques suggests strong potential for commercial adoption in the near future.

To gain an overview of the literature on lactate sensors, readers can refer to the review article by Ding et al. [99].

##### pH Sensors

pH is a measure of the acidity or alkalinity of a solution. Accurate pH measurement is fundamental to numerous environmental, biological, and chemical processes. Over time, a wide range of detection methods has been developed. Traditional pH sensing relies on potentiometric, chemiresistive, optical, mass-based, and capacitive techniques [100]. Typically, pH is measured using glass electrodes or ion-selective field-effect transistors (ISFETs). However, the mechanical rigidity of these electrodes, the need for a reference electrode, and the risk of electrolyte leakage present challenges for miniaturization and wearable applications on irregular surfaces [101].

As a result, newer strategies, particularly chemiresistive sensing, have gained prominence as low-cost, miniaturizable approaches suitable for wearable devices with minimal signal degradation. Monitoring pH levels in various bodily fluids, especially sweat, has received increasing attention because pH levels directly correlate with multiple health conditions. For instance, patients with type II diabetes or kidney stones typically have lower pH levels than healthy individuals [102], and many skin disorders are associated with abnormal pH values. Therefore, skin-mounted, noninvasive, in vivo, real-time pH monitoring is expected to play a crucial role in the early diagnosis of several medical conditions [103]. Human skin reflects changes in body pH, making it an ideal location for continuous monitoring.

Hydrated skin tends to be more acidic, while dehydrated skin becomes slightly more alkaline. Current research focuses on developing sensors that can distinguish between these physiological states. One example is an electrochemical device integrated with data acquisition and signal conditioning circuits for continuous, real-time monitoring of pH and calcium concentrations in body fluids [102]. The sensing results were validated using spectrometry and commercial pH meters, demonstrating high repeatability and selectivity. A capsule-sized, implantable pH sensor prototype was also developed for monitoring gastroesophageal reflux [104]. This system uses interdigitated electrodes for impedance and pH detection and operates wirelessly through external transponders.

Wearable and textile-based approaches have also been explored. For example, cotton-yarn-based conductive wire electrodes have been proposed for detecting pH and other analytes in the human body [105]. Carbon nanotubes (CNTs) served as the conductive filler and were coated with a polymeric membrane to form ion-selective electrodes. Functionalization plays a critical role in CNT-based sensing. Biofunctionalized, inkjet-printed CNTs have been demonstrated for pH detection [106], where the sensing mechanism relies on the doping and dedoping of CNTs by hydronium and hydroxide ions. Multiple printing cycles improved conductivity and ensured reproducible sensitivity and fast response times.

Screen-printed thick films of Ag/AgCl/KCl electrodes have been developed for simultaneous pH and temperature sensing [107]. Metal-oxide-based printed layers, such as screen-printed TiO_2_ thick films, have been investigated for pH measurement and water quality analysis [100]. In these devices, screen-printed interdigitated electrodes (IDEs) paired with TiO_2_ sensing layers exhibited impedance changes that were strongly correlated with pH variations. Recently, high-resolution aerosol-jet printing was used to fabricate CNT-based serpentine sensing layers with Ag electrodes [101]. The resulting miniature sensors exhibited rapid response times, high sensitivity, and promising biocompatibility for potential live-cell applications.

The same approaches used to create wearable pH sensors can be used for other cations, such as K^+^ and Na^+^. These cations are easily accessible in sweat and their concentrations can vary with certain illnesses [108]. One of the most relevant publications on this topic is the work of Pirovano et al. (2020). They developed a fully autonomous system called SwEatch that resembles a wristwatch, despite not being a Swatch^®^. They successfully tested the system with athletes. The Na^+^ and K^+^ sensors are potentiometric, of course. The Na^+^ ionophore was a calixarene and the K^+^ ionophore was valinomycin. More notable than the cation sensitivity, which is approximately +50 mV/dec for both ions, is the sensor architecture, which is based on poly (3-octylthiophene-2,5-diyl). This conductive polymer is used as the transducing component and is printed directly onto screen-printed carbon electrodes.

More recently, in 2024, Jalal et al. [109] used polyaniline for pH sensing and the two original inorganic ionophores, Na_0.44_MnO_2_ and K_2_Co[Fe(CN)_6_], for sensing Na^+^ and K^+^, respectively. Interestingly, no recent developments concerning the diagnosis of cystic fibrosis have been published. One of the most recent studies is that of De Matteis et al. [110], who investigated the electrochromic properties of tungsten oxide (WO_3_) in the presence of sodium. However, despite being developed to a demonstrator-grade level, none of these devices has reached the market yet.

**Figure 5:**
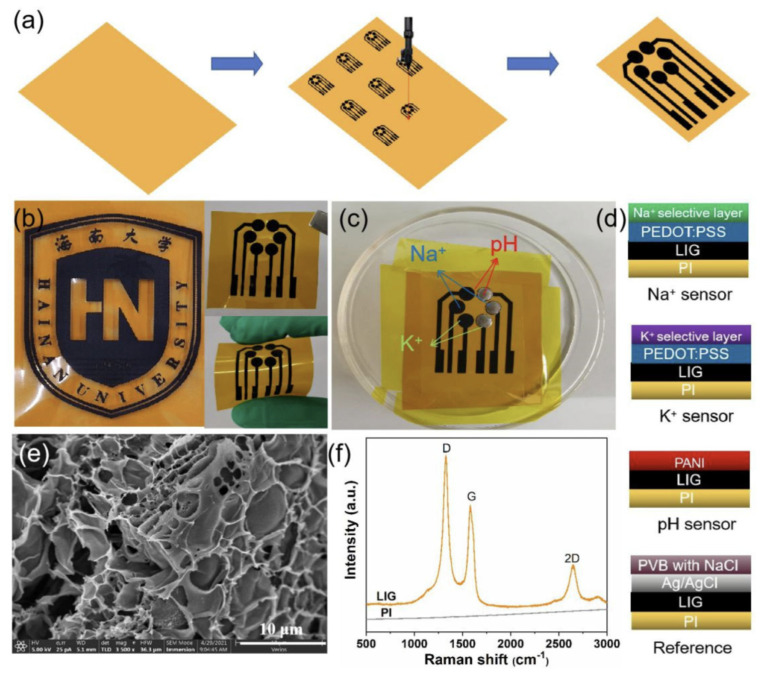
(**a**) Laser-Induced Graphene (LIG) electrodes obtained on polyimide flexible substrate. (**b**) Example of patterns obtained by LIG. (**c**) LIG sensors for detecting Na^+^, K^+^ and pH. (**d**) Operating schemes of the Na^+^, K^+^ and pH sensors, with their Ag/AgCl reference electrodes. (**e**) SEM image and (**f**) Raman spectra of the LIG layer. Reproduced from Liao et al. [111]. Open access figure distributed under the terms and conditions of the Creative Commons Attribution (CC BY) license.

Figure 5 shows an example of a flexible pH sensor coupled with sodium and potassium sensors. This sensor can be applied directly to the skin. It is produced by writing directly onto a polyimide substrate using the laser-induced graphene (LIG) technique.

For an overview of the literature on wearable pH sensors for wound monitoring, readers can refer to the review article by Li et al. [112].

Table 1 summarizes the sensing approaches, materials, substrates, and mechanisms used in fluid-based glucose, lactate, and pH sensing systems.

##### Cholesterol Sensors

Monitoring cholesterol levels is essential for maintaining human health because elevated levels are strongly correlated with an increased risk of cardiovascular and cerebrovascular diseases, including heart disease, stroke, hypertension, coronary artery disease, arteriosclerosis, and cerebral thrombosis [114]. Accordingly, substantial research efforts have focused on developing highly sensitive cholesterol biosensors that employ a variety of detection mechanisms. Recent advances in strategies to enhance the selectivity and sensitivity of enzymatic cholesterol sensors have been comprehensively reviewed elsewhere [115].

An integrated array of field-effect transistors (i-FETs) has been demonstrated for simultaneously and selectively detecting multiple analytes, specifically cholesterol, glucose, and urea [114]. In this system, ZnO nanorods serve as the sensing elements, improving device stability and enabling rapid multi-analyte analysis. Despite this progress, comparatively little research has addressed the fabrication of cholesterol sensors on polymeric substrates using printing-based manufacturing approaches. Wearable cholesterol-monitoring platforms are in the early stages of development and require substantially more research, particularly with respect to material selection and optimization of detection mechanisms.

For a comprehensive understanding of the literature on cholesterol monitoring systems, readers can refer to the review article by Rajan et al. [116].

### 2.2. Implantable Sensors

Implantable prototypes for healthcare applications are the second major type of wearable sensing system. These devices are inserted into the body to detect abnormalities or administer microdoses of therapeutic agents. Implantable sensors enable the continuous, real-time monitoring of internal biochemical variations and provide a direct interface for molecular transport and physiological signal tracking. Two primary categories of implantable technologies have been extensively investigated: neural sensors and drug-delivery systems. The most widely adopted examples are invasive glucose-monitoring devices, including blood glucose monitoring (BGM) and continuous glucose monitoring (CGM) systems. Several minimally invasive commercial CGM platforms, such as Abbott’s FreeStyle Libre 2 and Dexcom’s G6, quantify glucose levels in subcutaneous interstitial fluid. Despite their clinical utility, these devices can cause discomfort or pain during application.

Self-monitoring of blood glucose (SMBG) remains a common approach for assessing glucose at the point of care. This method requires obtaining a small blood sample via a finger prick and applying it to a reagent strip containing enzyme-based components, typically glucose oxidase. The enzymatic reaction generates an electrical signal that the SMBG device subsequently processes to yield an accurate glucose concentration. SMBG systems are valued for their simplicity, reliability, and usefulness in daily glucose management. However, they only provide single-time-point measurements and therefore fail to capture the full spectrum of glucose fluctuations occurring over a 24-h period. Furthermore, repeated finger pricking can cause discomfort and reduce patient adherence to regular monitoring [117]. One example is the FreeStyle Precision Neo meter by Abbott, a compact device that can analyze extremely small blood volumes in seconds. It is equipped with integrated data storage and trend analysis features.

Continuous glucose monitoring (CGM) systems are a more advanced solution that employ a subcutaneous sensor to deliver real-time glucose data at frequent intervals. This continuous data stream gives users a comprehensive view of diurnal glucose dynamics and enables earlier detection of hyperglycemic or hypoglycemic events. CGM is particularly beneficial for individuals with highly variable glycemic profiles because it offers substantially more information than SMBG. However, adoption is limited by the high cost of the technology, and device accuracy may decline during rapid glucose fluctuations, which can influence clinical decision-making [118]. The Dexcom G6 system is an example of current CGM technology. It features a subcutaneous sensor with a functional lifespan of up to 10 days, as well as wireless transmission of real-time glucose readings to smartphones or other devices. Despite these advantages, accessibility and affordability remain significant challenges, underscoring the need for further innovation.

Although invasive approaches such as SMBG and CGM are considered the clinical gold standard, they have several drawbacks. Frequent skin penetration or prolonged sensor implantation can result in pain, irritation, and user fatigue. Improper sterilization, reuse of lancets, or sharing of monitoring equipment can also increase the risk of transmitting bloodborne pathogens, especially in environments where disposable medical supplies are limited [119]. These limitations have intensified interest in developing non-invasive glucose-sensing technologies that can provide accurate, continuous measurements without causing physical harm. Such innovations would substantially enhance patient comfort while mitigating the safety risks associated with traditional invasive methods. Over the past few decades, significant research efforts have focused on advancing various non-invasive strategies for continuous glucose monitoring.

Implantable monitoring systems have been reviewed; for example, see the work of Yu et al. [120].

## 3. Mature Applications of Health Monitoring Sensors

Several health-monitoring sensor technologies have reached maturity and stability. They have been widely adopted and extensively validated for routine use in clinical and consumer settings. These technologies are listed in Table 2.

Cardiovascular monitoring is one of the oldest and most reliable application areas. Electrocardiography and heart rate monitoring, implemented through Holter monitors, chest straps, and smartwatches, are well-established for diagnostic and longitudinal monitoring purposes. Blood pressure monitoring using oscillometric cuffs remains the clinical reference standard. However, some cuffless blood pressure measurement methods have now reached a sufficient level of maturity and validation for broader deployment. Pulse oximeters that measure peripheral oxygen saturation (SpO_2_) are widely used in hospitals and are increasingly being integrated into wearable devices.

Glucose monitoring is another area with highly developed sensor technologies. Continuous glucose monitors (CGMs), such as those developed by Dexcom and Abbott FreeStyle Libre, are FDA-approved and widely used in diabetes management. These systems continuously measure glucose levels and have become integral to modern clinical practice for both type 1 and type 2 diabetes.

Technologies that track activity and motion using accelerometers and gyroscopes are also well established. These sensors are commonly used for counting steps, detecting falls, monitoring sleep, and tracking sedentary behavior. They are commonly embedded in fitness bands, smartwatches, and medical-grade wearables used for clinical monitoring and functional assessment.

Respiratory monitoring has a long history in clinical care and has evolved to include wearable technology. Impedance-based respiration rate sensors and chest-worn bands are widely used, and capnography remains a standard technique in clinical settings, particularly in anesthesia and intensive care. Wearable respiratory belts are now used for continuous respiratory monitoring at home and in hospitals.

Similarly, temperature monitoring technologies are mature. Digital skin temperature sensors and ingestible temperature sensors have long been used in clinical and research settings. More recently, continuous temperature patches have been introduced for fever tracking, fertility monitoring, and long-term physiological monitoring.

Sleep monitoring is a well-established field with applications in both the clinical and consumer sectors. Polysomnography, which incorporates electroencephalography (EEG), electromyography (EMG), airflow sensors, SpO_2_ measurement, and thoracic effort sensors, is a fully mature technology and remains the clinical gold standard. Meanwhile, wearable sleep trackers, such as Fitbit and Oura, have also matured and are widely used for sleep monitoring outside of clinical laboratories.

Implantable sensors are among the most advanced health-monitoring technologies. Cardiac pacemakers and implantable cardiac monitors have decades of proven clinical use and provide continuous cardiac sensing and therapy. Similarly, cochlear implants are a long-established class of implantable sensor systems with extensive clinical validation.

Mature sensing solutions have also benefited gait and rehabilitation monitoring. Inertial measurement units (IMUs) are widely used for gait analysis and tracking progress in physical therapy and rehabilitation, and have been broadly adopted in sports medicine and rehabilitation settings.

Finally, environmental and physiological stress sensors have achieved stable commercial deployment. Skin conductance sensors that measure electrodermal activity (EDA) are used for stress monitoring in mature products, such as the Empatica E4. UV exposure sensors are also being integrated into wearable devices to monitor environmental exposure.

These applications represent a set of health-monitoring sensor technologies characterized by technical stability, clinical validation, regulatory acceptance, and large-scale use in clinical practice and consumer health monitoring.

## 4. Conclusions: Prospects and Challenges

Wearable sensors, such as photoplethysmography (PPG) watches, inertial measurement units (IMUs), electrocardiogram (ECG) patches, smart textiles, and biochemical sensors, have become central to modern digital health. Advances in miniaturized electronics, wireless communication, ultra-low-power systems, and AI-based analytics have driven their rapid development. The literature widely recognizes the potential of wearables for continuous health monitoring, remote patient management, and preventive medicine. However, it also emphasizes significant methodological, regulatory, and ethical challenges.

Wearables enable the persistent monitoring of physiological and behavioral parameters, including heart rate, respiration, physical activity, and sleep, in settings beyond traditional clinical environments. Continuous data acquisition supports longitudinal tracking and the early detection of physiological deviations, enabling earlier intervention than episodic clinical measurements [121,122]. Machine learning methods applied to wearable sensor data can identify subtle trends that predict disease onset, including cardiac arrhythmias, sleep disorders, metabolic dysregulation, and stress-related conditions [123,124].

Wearable technologies have demonstrated their clinical usefulness in managing chronic diseases. For example, continuous glucose monitoring can help manage diabetes, connected blood-pressure cuffs can help manage hypertension, oxygen monitoring can help manage chronic obstructive pulmonary disease, and ECG-based devices can help manage cardiac arrhythmias [125,126]. Furthermore, AI-driven data fusion techniques can integrate multimodal sensor streams to derive personalized risk scores and digital biomarkers that capture individual physiological trajectories [127,128]. Clinical-grade wearables also support decentralized healthcare models by enabling home-based monitoring, reducing the burden on hospitals, and facilitating remote clinical decision-making [129,130].

Despite these advantages, several limitations remain. Consumer-grade wearables are often affected by motion artifacts, variable skin perfusion, optical interference, and algorithmic inconsistencies. These factors result in reduced accuracy compared with that of gold-standard clinical equipment [131,132]. The heterogeneity of sensing modalities, signal-processing algorithms, calibration procedures, and data formats across devices is a recurring challenge that limits interoperability, reproducibility, and large-scale data aggregation [133,134].

Wearables generate intimate, high-resolution biometric data, raising concerns related to re-identification risks, cybersecurity vulnerabilities, algorithmic bias, and the potential misuse of health data by employers or insurers [135,136]. From an engineering perspective, trade-offs between user comfort, sampling frequency, sensor quality, and battery capacity remain major constraints. Long-term user adherence is often low, and data accuracy depends heavily on proper device placement and maintenance [137,138].

From a regulatory standpoint, medical-grade wearables must undergo rigorous evaluation under FDA or CE frameworks, which can significantly impede innovation and market adoption. In contrast, most consumer devices lack medical certification and are not reliable for clinical decision-making [139]. Additionally, clinicians face challenges integrating high volumes of wearable data into electronic health record systems without contributing to alert fatigue or an increased cognitive burden [140].

Overall, the literature agrees that wearable sensors have the potential to transform healthcare by shifting it from a reactive model to a preventive, continuous, and personalized one. Their ability to generate rich longitudinal datasets surpasses that of traditional clinical tools. However, challenges related to accuracy, standardization, privacy, regulation, and clinical integration must be addressed to enable their safe and effective adoption in mainstream healthcare. Indeed, the clinical translation of healthcare wearable sensors depends on more than just technical performance. It also requires robust frameworks for privacy, fairness, security, consent, and interoperability. The continuous and highly granular collection of data poses significant privacy risks and can create algorithmic bias if the datasets are not representative. This underscores the importance of privacy-by-design principles and systematic bias assessments. Strong data security measures, including end-to-end encryption and clear data governance, are essential to maintaining trust and protecting sensitive health information. Adaptive consent models that reflect the ongoing and evolving use of wearable data are equally important. Finally, interoperability standards, such as FHIR (Fast Healthcare Interoperability Resources), are critical for integrating wearable data into clinical workflows and electronic health records. This will enable the data to be used in a scalable, reproducible, and clinically meaningful way.

## Figures and Tables

**Table 1:** Summary of representative materials, substrates, sensing mechanisms, and fabrication procedures used in fluidic-based sensor systems [113].

Sensor Type	Materials	Substrates	Mechanism	Fabrication	References
		Electrode Area	Linear Range	LOD or Sensitivity	
Glucose sensors	Ag/AgCl	Polyurethane patch	Iontophoresis + amperometry	Screen printing	[79]
		Centimetric	0–100 μM	Not given	
	Cr/Au, Cr/Pt	PI/PDMS	Amperometry	Transfer printing	[82]
		Millimetric	0–0.8 mM	Not given	
	In_2_O_3_	PET	FET	Shadow masking	[81]
		ca. 500 × 500 μm	0.1–100 μM	10 nM	
	Ag/AgCl	PET	Iontophoresis + amperometry	Lithography	[76]
		Centimetric	0–150 μM	Not given	
	Ag/AgCl, PEDOT-PSS	PET	Amperometric	Vacuum deposition	[78]
		Centimetric	-	-	
	rGO, AuNPs, PtNPs	PI	Amperometric	E-beam evaporation	[84]
		Centimetric	0.1–2.3 mM	Not given	
Lactate sensors	Carbon, Ag/AgCl	Textile (cotton)	Amperometric	Screen printing	[83]
		Centimetric	0.05–1.5 mM	0.05 mM	
	Carbon, Ag/AgCl	Polymeric tubes	Amperometric	Manual printing	[92]
		Centimetric	0.1–1 mM	85 μM	
	Graphene oxide, Ag/AgCl	Plastic patch	Electrochemical	Screen printing	[88]
		Centimetric	0–20 mM	1 mM	
	MWCNTs, Ag/AgCl	Paper (Papilio)	Electrochemical	Screen printing	[88]
		Centimetric	0–20 mM	Not given	
	Ag/AgCl	PET	Iontophoresis + amperometry	Lithography	[80]
		Centimetric	0–30 mM	Not given	
	NiO	PET	Potentiometric	Screen printing	[89]
		Centimetric	0.2–3 mM	2 μM	
pH sensors	CNT, Ag	PI	Conductimetric	Aerosol jet printing	[101]
		Centimetric	pH 4–pH 10	10% pH^−1^	
	SWCTs	Liquid crystal polymer	Conductimetric	Inkjet, Screen printing	[102]
		Centimetric	pH 3–pH 8	63 mV pH^−1^	
	SWCNTs	Cotton yarn	Potentiometric	Screen printing	[105]
		Millimetric	pH 2–pH 11	59 mV pH^−1^	
	PVB-Ag/AgCl, PANI	PET	Potentiometric	Electrochemical deposition	[103]
		Centimetric	pH 4–pH 8	71 mV pH^−1^	
K^+^ and Na^+^ sensors	Poly (3-octylthiophene-2,5-diyl), Carbon SPE	PET	Potentiometric	Automated pipetting	[108]
		Millimetric	10^−4^–10^−1^ M for Na^+^ and K^+^	52 mV pH^−1^ for Na^+^ and 46 mV pH^−1^ for K^+^	
	Na_0.44_MnO_2_, polyaniline, and K_2_Co[Fe(CN)_6_]	PVC	Potentiometric	Sputtering, Drop-casting	[109]
		Centimetric	0–2 mM	59 mV pH^−1^ for Na^+^ and K^+^	
	WO_3_	ITO/glass	Transmittance	Electron beam	[110]
		Centimetric	30–120 mM Na^+^	50% at 555 nm for 120 mM Na^+^	

**Table 2:** Mature Health-Sensor Applications with Example Devices.

Sensor/Application	Wellness Devices	Clinical Devices
Heart rate/ECG	e.g., Samsung Galaxy Fit 3: a fitness tracker that monitors heart rate, steps, sleep, general activity	Clinical ambulatory ECG monitors (patches or Holter devices) for rhythm monitoring
Blood Oxygen (SpO_2_)	e.g., One Medical Oxy One Pro: a fingertip pulse oximeter available to general consumers	Hospital-grade pulse oximeters used for respiratory monitoring
Activity/Steps/Movement	e.g., Amazfit Helio Strap: wearable tracker for steps, activity, sleep, daily movement	Clinical-grade activity/rehabilitation trackers (e.g., inertial measurement units in gait labs) for rehab, mobility assessment
Sleep Tracking	Many smartwatches or fitness bands (e.g., via accelerometer + heart-rate) for general sleep patterns	Clinical-grade sleep monitoring systems (polysomnography) for sleep disorder diagnosis
Blood Pressure (Home Monitoring)	Some smartwatches/wrist devices attempt BP estimation (requires calibration)	Dedicated home blood-pressure monitors (arm cuffs) or clinic sphygmomanometers—standard medical devices
Continuous Glucose Monitoring (CGM)	Consumer-grade CGM still limited	Dexcom CGM—widely used by people with diabetes to continuously monitor interstitial glucose
Respiratory Rate/Oxygenation (for sleep apnea, COPD, lung disease)	Some wearables attempt estimation of breathing rate, but with limited accuracy	Clinical respiratory monitors, pulse oximeters, capnography used in hospitals or at-home under supervision
Skin Temperature/Body Temperature	Smart thermometers or wearable “wellness” patches (non-invasive)	Medical-grade temperature monitors for fever tracking/hospital use (often part of vital-sign monitoring devices)
Fall Detection/Emergency Alert	Smart bracelets/watches with accelerometer + gyroscope + fall detection features	Clinical alert systems (sometimes combined with ECG/SpO_2_ monitors) for elderly care or chronic disease management
ECG Holter/Extended Cardiac Monitoring	Some “semi-consumer” portable ECG devices (more for wellness or preliminary screening)	Full Holter monitors, ambulatory ECG patches—gold standard for arrhythmia diagnosis
Remote Patient Monitoring (multi-parametric: HR, SpO_2_, ECG, etc.)	Limited—some connected wearables with several sensors, but generally non-medical	Hospital-at-home devices, “vital-sign monitors” used in telemedicine/home care settings
Smart Clothing/Textile Sensors	Emerging: sensor-embedded garments for activity, basic vital signs (accelerometer, PPG, etc.)	Smart textile systems used in clinical monitoring or rehabilitation for continuous monitoring

## Data Availability

Data sharing is not applicable.

## Abbreviations

The following abbreviations are used in this manuscript:

Ag/AgCl	Silver/Silver Chloride
BGM	Blood Glucose Monitoring
CNT	Carbon Nanotube
COPD	Chronic Obstructive Pulmonary Disease
CVD	Cardiovascular Disease
DN	Double Network
ECG	Electrocardiogram
FP	Follicular Phase
IMU	Inertial Measurement Unit
ISF	Interstitial Fluid
LBL	Layer By Layer
LP	Luteal Phase
PDMS	Polydimethylsiloxane
PPG	Photoplethysmography
PVDF-TrFE	Poly(vinylidene fluoride-co-trifluoroethylene)
RR	R-to-R interval
SMBG	Self-Monitoring of Blood Glucose
SpO_2_	Blood Oxygen Saturation
SWCNT	Single-Walled Carbon Nanotube
TCR	Thermal Coefficient of Resistance
WHO	World Health Organization

## References

[1] Kim J., Campbell A.S., De Ávila B.E.-F., Wang J. (2019). Wearable Biosensors for Healthcare Monitoring. Nat. Biotechnol..

[2] Khan Y., Ostfeld A.E., Lochner C.M., Pierre A., Arias A.C. (2016). Monitoring of Vital Signs with Flexible and Wearable Medical Devices. Adv. Mater..

[3] Nassar J.M., Mishra K., Lau K., Aguirre-Pablo A.A., Hussain M.M. (2017). Recyclable Nonfunctionalized Paper-Based Ultralow-Cost Wearable Health Monitoring System. Adv. Mater. Technol..

[4] Wang Y., Wang L., Yang T., Li X., Zang X., Zhu M., Wang K., Wu D., Zhu H. (2014). Wearable and Highly Sensitive Graphene Strain Sensors for Human Motion Monitoring. Adv. Funct. Mater..

[5] World Health Organization (2011). Global Atlas on Cardiovascular Disease Prevention and Control.

[6] Myerburg R.J., Junttila M.J. (2012). Sudden Cardiac Death Caused by Coronary Heart Disease. Circulation.

[7] Chen S., Qi J., Fan S., Qiao Z., Yeo J.C., Lim C.T. (2021). Flexible Wearable Sensors for Cardiovascular Health Monitoring. Adv. Healthc. Mater..

[8] Haji S.A., Movahed A. (2000). Right Ventricular Infarction—Diagnosis and Treatment. Clin. Cardiol..

[9] O’Rourke M.F., Mancia G. (1999). Arterial Stiffness. J. Hypertens..

[10] Stergiou G.S., Alpert B., Mieke S., Asmar R., Atkins N., Eckert S., Frick G., Friedman B., Graßl T., Ichikawa T. (2018). A Universal Standard for the Validation of Blood Pressure Measuring Devices: Association for the Advancement of Medical Instrumentation/European Society of Hypertension/International Organization for Standardization (AAMI/ESH/ISO) Collaboration Statement. Hypertension.

[11] Islam M.S. (2017). Hypertension.

[12] Kireev D., Sel K., Ibrahim B., Kumar N., Akbari A., Jafari R., Akinwande D. (2022). Continuous Cuffless Monitoring of Arterial Blood Pressure via Graphene Bioimpedance Tattoos. Nat. Nanotechnol..

[13] Peng C., Chen M., Sim H.K., Zhu Y., Jiang X. (2021). Noninvasive and Nonocclusive Blood Pressure Monitoring via a Flexible Piezo-Composite Ultrasonic Sensor. IEEE Sens. J..

[14] Chi Y.M., Jung T.-P., Cauwenberghs G. (2010). Dry-Contact and Noncontact Biopotential Electrodes: Methodological Review. IEEE Rev. Biomed. Eng..

[15] Oresko J.J., Jin Z., Cheng J., Huang S., Sun Y., Duschl H., Cheng A.C. (2010). A Wearable Smartphone-Based Platform for Real-Time Cardiovascular Disease Detection Via Electrocardiogram Processing. IEEE Trans. Inf. Technol. Biomed..

[16] Eleyan A., AlBoghbaish E., AlShatti A., AlSultan A., AlDarbi D. (2024). RHYTHMI: A Deep Learning-Based Mobile ECG Device for Heart Disease Prediction. Appl. Syst. Innov..

[17] Meziane N., Webster J.G., Attari M., Nimunkar A.J. (2013). Dry Electrodes for Electrocardiography. Physiol. Meas..

[18] Huang Y., Song Y., Gou L., Zou Y. (2021). A Novel Wearable Flexible Dry Electrode Based on Cowhide for ECG Measurement. Biosensors.

[19] Lochner C.M., Khan Y., Pierre A., Arias A.C. (2014). All-Organic Optoelectronic Sensor for Pulse Oximetry. Nat. Commun..

[20] Sim J.K., Ahn B., Doh I. (2018). A Contact-Force Regulated Photoplethysmography (PPG) Platform. AIP Adv..

[21] Wang C., Li X., Hu H., Zhang L., Huang Z., Lin M., Zhang Z., Yin Z., Huang B., Gong H. (2018). Monitoring of the Central Blood Pressure Waveform via a Conformal Ultrasonic Device. Nat. Biomed. Eng..

[22] Kang S., Pradana Rachim V., Baek J.-H., Lee S.Y., Park S.-M. (2020). A Flexible Patch-Type Strain Sensor Based on Polyaniline for Continuous Monitoring of Pulse Waves. IEEE Access.

[23] Wang J., Zhu Y., Wu Z., Zhang Y., Lin J., Chen T., Liu H., Wang F., Sun L. (2022). Wearable Multichannel Pulse Condition Monitoring System Based on Flexible Pressure Sensor Arrays. Microsyst. Nanoeng..

[24] Sekine T., Gaïtis A., Sato J., Miyazawa K., Muraki K., Shiwaku R., Takeda Y., Matsui H., Kumaki D., Domingues Dos Santos F. (2019). Low Operating Voltage and Highly Pressure-Sensitive Printed Sensor for Healthcare Monitoring with Analogic Amplifier Circuit. ACS Appl. Electron. Mater..

[25] Tan P., Xi Y., Chao S., Jiang D., Liu Z., Fan Y., Li Z. (2022). An Artificial Intelligence-Enhanced Blood Pressure Monitor Wristband Based on Piezoelectric Nanogenerator. Biosensors.

[26] Prieto-Avalos G., Cruz-Ramos N.A., Alor-Hernández G., Sánchez-Cervantes J.L., Rodríguez-Mazahua L., Guarneros-Nolasco L.R. (2022). Wearable Devices for Physical Monitoring of Heart: A Review. Biosensors.

[27] Zhang S., Wang S., Wang Y., Fan X., Ding L., Xuan S., Gong X. (2018). Conductive Shear Thickening Gel/Polyurethane Sponge: A Flexible Human Motion Detection Sensor with Excellent Safeguarding Performance. Compos. Part Appl. Sci. Manuf..

[28] Yadav A., Yadav N., Wu Y., RamaKrishna S., Hongyu Z. (2023). Wearable strain sensors: State-of-the-art and future applications. Mater. Adv..

[29] Li Q., Li J., Tran D., Luo C., Gao Y., Yu C., Xuan F. (2017). Engineering of Carbon Nanotube/Polydimethylsiloxane Nanocomposites with Enhanced Sensitivity for Wearable Motion Sensors. J. Mater. Chem. C.

[30] Menguc Y., Park Y.-L., Martinez-Villalpando E., Aubin P., Zisook M., Stirling L., Wood R.J., Walsh C.J. (2013). Soft Wearable Motion Sensing Suit for Lower Limb Biomechanics Measurements. Proceedings of the 2013 IEEE International Conference on Robotics and Automation.

[31] Liu L., Liang X., Wan X., Kuang X., Zhang Z., Jiang G., He H. (2023). A review on knitted flexible strain sensors for human activity monitoring. Adv. Mater. Technol..

[32] Yogeswaran N., Dang W., Navaraj W.T., Shakthivel D., Khan S., Polat E.O., Gupta S., Heidari H., Kaboli M., Lorenzelli L. (2015). New Materials and Advances in Making Electronic Skin for Interactive Robots. Adv. Robot..

[33] Dahiya R., Navaraj W.T., Khan S., Polat E.O. (2015). Developing Electronic Skin with the Sense of Touch. Inf. Disp..

[34] Kim D., Kwon J., Han S., Park Y.-L., Jo S. (2019). Deep Full-Body Motion Network for a Soft Wearable Motion Sensing Suit. IEEE/ASME Trans. Mechatron..

[35] Mengüç Y., Park Y.-L., Pei H., Vogt D., Aubin P.M., Winchell E., Fluke L., Stirling L., Wood R.J., Walsh C.J. (2014). Wearable Soft Sensing Suit for Human Gait Measurement. Int. J. Robot. Res..

[36] Boland C.S., Khan U., Backes C., O’Neill A., McCauley J., Duane S., Shanker R., Liu Y., Jurewicz I., Dalton A.B. (2014). Sensitive, High-Strain, High-Rate Bodily Motion Sensors Based on Graphene–Rubber Composites. ACS Nano.

[37] Yao S., Zhu Y. (2014). Wearable Multifunctional Sensors Using Printed Stretchable Conductors Made of Silver Nanowires. Nanoscale.

[38] Li C., Cui Y.-L., Tian G.-L., Shu Y., Wang X.-F., Tian H., Yang Y., Wei F., Ren T.-L. (2015). Flexible CNT-Array Double Helices Strain Sensor with High Stretchability for Motion Capture. Sci. Rep..

[39] Amjadi M., Yoon Y.J., Park I. (2015). Ultra-Stretchable and Skin-Mountable Strain Sensors Using Carbon Nanotubes–Ecoflex Nanocomposites. Nanotechnology.

[40] Christ J.F., Aliheidari N., Ameli A., Pötschke P. (2017). 3D Printed Highly Elastic Strain Sensors of Multiwalled Carbon Nanotube/Thermoplastic Polyurethane Nanocomposites. Mater. Des..

[41] Khan S., Lorenzelli L., Dahiya R.S. (2014). Bendable Piezoresistive Sensors by Screen Printing MWCNT/PDMS Composites on Flexible Substrates. Proceedings of the 2014 10th Conference on Ph.D. Research in Microelectronics and Electronics (PRIME).

[42] Zhang H., Chen X., Liu Y., Yang C., Liu W., Qi M., Zhang D. (2024). PDMS film-based flexible pressure sensor array with surface protruding structure for human motion detection and wrist posture recognition. ACS Appl. Mater. Interfaces.

[43] Li Y.-Q., Huang P., Zhu W.-B., Fu S.-Y., Hu N., Liao K. (2017). Flexible Wire-Shaped Strain Sensor from Cotton Thread for Human Health and Motion Detection. Sci. Rep..

[44] Yamamoto Y., Harada S., Yamamoto D., Honda W., Arie T., Akita S., Takei K. (2016). Printed Multifunctional Flexible Device with an Integrated Motion Sensor for Health Care Monitoring. Sci. Adv..

[45] Xie R., Xie Y., López-Barrón C.R., Gao K.-Z., Wagner N.J. (2017). Ultra-Stretchable Conductive Iono-Elastomer and Motion Strain Sensor System Developed Therefrom. Technol. Innov..

[46] Zhou L.-Y., Gao Q., Zhan J.-F., Xie C.-Q., Fu J.-Z., He Y. (2018). Three-Dimensional Printed Wearable Sensors with Liquid Metals for Detecting the Pose of Snakelike Soft Robots. ACS Appl. Mater. Interfaces.

[47] Chang Z., Gao J., Liu X., Li Z., Zhang J., Zhang J., Zhang L., Wang T., Wang H., Li M. (2025). Recent Advances and Future Prospects in Wearable Flexible Sensors for Motion Monitoring. Bio Integr..

[48] Dolson C.M., Harlow E.R., Phelan D.M., Gabbett T.J., Gaal B., McMellen C., Geletka B.J., Calcei J.G., Voos J.E., Seshadri D.R. (2022). Wearable sensor technology to predict core body temperature: A systematic review. Sensors.

[49] Harada S., Kanao K., Yamamoto Y., Arie T., Akita S., Takei K. (2014). Fully Printed Flexible Fingerprint-like Three-Axis Tactile and Slip Force and Temperature Sensors for Artificial Skin. ACS Nano.

[50] Harada S., Honda W., Arie T., Akita S., Takei K. (2014). Fully Printed, Highly Sensitive Multifunctional Artificial Electronic Whisker Arrays Integrated with Strain and Temperature Sensors. ACS Nano.

[51] Khan S., Nguyen T.P., Thiery L., Vairac P., Briand D. (2017). Aerosol Jet Printing of Miniaturized, Low Power Flexible Micro-Hotplates. Proceedings.

[52] Ali S., Hassan A., Bae J., Lee C.H., Kim J. (2016). All-Printed Differential Temperature Sensor for the Compensation of Bending Effects. Langmuir.

[53] Tao X., Liao S., Wang S., Wu D., Wang Y. (2018). Body Compatible Thermometer Based on Green Electrolytes. ACS Sens..

[54] Vena A., Sydanheimo L., Tentzeris M.M., Ukkonen L. (2015). A Fully Inkjet-Printed Wireless and Chipless Sensor for CO_2_ and Temperature Detection. IEEE Sens. J..

[55] Chen Y., Lu B., Chen Y., Feng X. (2015). Breathable and Stretchable Temperature Sensors Inspired by Skin. Sci. Rep..

[56] Vuorinen T., Niittynen J., Kankkunen T., Kraft T.M., Mäntysalo M. (2016). Inkjet-Printed Graphene/PEDOT:PSS Temperature Sensors on a Skin-Conformable Polyurethane Substrate. Sci. Rep..

[57] Childs A., Mayol B., Lasalde-Ramírez J.A., Song Y., Sempionatto J.R., Gao W. (2024). Diving into sweat: Advances, challenges, and future directions in wearable sweat sensing. ACS Nano.

[58] Nakata S., Arie T., Akita S., Takei K. (2017). Wearable, Flexible, and Multifunctional Healthcare Device with an ISFET Chemical Sensor for Simultaneous Sweat pH and Skin Temperature Monitoring. ACS Sens..

[59] Liu G., Tan Q., Kou H., Zhang L., Wang J., Lv W., Dong H., Xiong J. (2018). A Flexible Temperature Sensor Based on Reduced Graphene Oxide for Robot Skin Used in Internet of Things. Sensors.

[60] Zhang H., Zhao K., Cui S., Yang J., Zhou D., Tang L., Shen J., Feng S., Zhang W., Fu Y. (2018). Anomalous Temperature Coefficient of Resistance in Graphene Nanowalls/Polymer Films and Applications in Infrared Photodetectors. Nanophotonics.

[61] Kim S., Oh S., Jung Y., Moon H., Lim H. (2018). Customizable, Flexible Pressure, and Temperature Step Sensors with Human Skinlike Color. ACS Omega.

[62] Oh J.H., Hong S.Y., Park H., Jin S.W., Jeong Y.R., Oh S.Y., Yun J., Lee H., Kim J.W., Ha J.S. (2018). Fabrication of High-Sensitivity Skin-Attachable Temperature Sensors with Bioinspired Microstructured Adhesive. ACS Appl. Mater. Interfaces.

[63] Wu J., Han S., Yang T., Li Z., Wu Z., Gui X., Tao K., Miao J., Norford L.K., Liu C. (2018). Highly Stretchable and Transparent Thermistor Based on Self-Healing Double Network Hydrogel. ACS Appl. Mater. Interfaces.

[64] Stier A., Halekote E., Mark A., Qiao S., Yang S., Diller K., Lu N. (2018). Stretchable Tattoo-Like Heater with On-Site Temperature Feedback Control. Micromachines.

[65] Li P., Zhou J., Cui Y., Ouyang J., Su Z., Zou Y., Liang J., Wang F., He K., Liu Y. (2025). A scalable, robust and high-sensitivity fiber sensor for real-time body temperature monitoring. Soft Sci..

[66] Hashimoto Y. (2026). A Comprehensive Review of Non-Invasive Core Body Temperature Measurement Techniques. Preprints.

[67] Nwibor C., Haxha S., Ali M.M., Sakel M., Haxha A.R., Saunders K., Nabakooza S. (2023). Remote Health Monitoring System for the Estimation of Blood Pressure, Heart Rate, and Blood Oxygen Saturation Level. IEEE Sens. J..

[68] Dcosta J.V., Ochoa D., Sanaur S. (2023). Recent progress in flexible and wearable all organic photoplethysmography sensors for SpO_2_ monitoring. Adv. Sci..

[69] Roglic G., World Health Organization (2016). Global Report on Diabetes.

[70] Ogurtsova K., Da Rocha Fernandes J.D., Huang Y., Linnenkamp U., Guariguata L., Cho N.H., Cavan D., Shaw J.E., Makaroff L.E. (2017). IDF Diabetes Atlas: Global Estimates for the Prevalence of Diabetes for 2015 and 2040. Diabetes Res. Clin. Pract..

[71] Clark L.C., Lyons C. (1962). Electrode Systems for Continuous Monitoring in Cardiovascular Surgery. Ann. N. Y. Acad. Sci..

[72] Bruen D., Delaney C., Florea L., Diamond D. (2017). Glucose Sensing for Diabetes Monitoring: Recent Developments. Sensors.

[73] Lee H., Hong Y.J., Baik S., Hyeon T., Kim D. (2018). Enzyme-Based Glucose Sensor: From Invasive to Wearable Device. Adv. Healthc. Mater..

[74] Steinberg M.D., Kassal P., Steinberg I.M. (2016). System Architectures in Wearable Electrochemical Sensors. Electroanalysis.

[75] Yu Q., Boussaid F., Bermak A., Tsui C.-Y. (2018). Room-Temperature Dual-Mode CMOS Gas-FET Sensor for Diabetes Detection. Proceedings of the 2018 IEEE International Symposium on Circuits and Systems (ISCAS).

[76] Kim J., Sempionatto J.R., Imani S., Hartel M.C., Barfidokht A., Tang G., Campbell A.S., Mercier P.P., Wang J. (2018). Simultaneous Monitoring of Sweat and Interstitial Fluid Using a Single Wearable Biosensor Platform. Adv. Sci..

[77] Yoon H., Xuan X., Jeong S., Park J.Y. (2018). Wearable, Robust, Non-Enzymatic Continuous Glucose Monitoring System and Its in Vivo Investigation. Biosens. Bioelectron..

[78] Aleeva Y., Maira G., Scopelliti M., Vinciguerra V., Scandurra G., Cannata G., Giusi G., Ciofi C., Figa V., Occhipinti L.G. (2018). Amperometric Biosensor and Front-End Electronics for Remote Glucose Monitoring by Crosslinked PEDOT-Glucose Oxidase. IEEE Sens. J..

[79] Bandodkar A.J., Jia W., Yardımcı C., Wang X., Ramirez J., Wang J. (2015). Tattoo-Based Noninvasive Glucose Monitoring: A Proof-of-Concept Study. Anal. Chem..

[80] Gao W., Emaminejad S., Nyein H.Y.Y., Challa S., Chen K., Peck A., Fahad H.M., Ota H., Shiraki H., Kiriya D. (2016). Fully Integrated Wearable Sensor Arrays for Multiplexed in Situ Perspiration Analysis. Nature.

[81] Liu Q., Liu Y., Wu F., Cao X., Li Z., Alharbi M., Abbas A.N., Amer M.R., Zhou C. (2018). Highly Sensitive and Wearable In_2_O_3_ Nanoribbon Transistor Biosensors with Integrated On-Chip Gate for Glucose Monitoring in Body Fluids. ACS Nano.

[82] Lee H., Song C., Hong Y.S., Kim M., Cho H.R., Kang T., Shin K., Choi S.H., Hyeon T., Kim D.-H. (2017). Wearable/Disposable Sweat-Based Glucose Monitoring Device with Multistage Transdermal Drug Delivery Module. Sci. Adv..

[83] Luo X., Yu H., Cui Y. (2018). A Wearable Amperometric Biosensor on a Cotton Fabric for Lactate. IEEE Electron. Device Lett..

[84] Xuan X., Yoon H.S., Park J.Y. (2018). A Wearable Electrochemical Glucose Sensor Based on Simple and Low-Cost Fabrication Supported Micro-Patterned Reduced Graphene Oxide Nanocomposite Electrode on Flexible Substrate. Biosens. Bioelectron..

[85] Chen J., Tao X., Xu X., Sun L., Huang R., Nilghaz A., Tian J. (2024). Making commercial bracelet smarter with a biochemical button module. Biosens. Bioelectron..

[86] Xue Y., Thalmayer A.S., Zeising S., Fischer G., Lübke M. (2022). Commercial and scientific solutions for blood glucose monitoring—A review. Sensors.

[87] Rassaei L., Olthuis W., Tsujimura S., Sudhölter E.J.R., Van Den Berg A. (2014). Lactate Biosensors: Current Status and Outlook. Anal. Bioanal. Chem..

[88] Jia W., Bandodkar A.J., Valdés-Ramírez G., Windmiller J.R., Yang Z., Ramírez J., Chan G., Wang J. (2013). Electrochemical Tattoo Biosensors for Real-Time Noninvasive Lactate Monitoring in Human Perspiration. Anal. Chem..

[89] Chou J.-C., Yan S.-J., Liao Y.-H., Lai C.-H., Wu Y.-X., Wu C.-Y., Chen H.-Y., Huang H.-Y., Wu T.-Y. (2017). Fabrication of Flexible Arrayed Lactate Biosensor Based on Immobilizing LDH-NAD+ on NiO Film Modified by GO and MBs. Sensors.

[90] Tuteja S.K., Ormsby C., Neethirajan S. (2018). Noninvasive Label-Free Detection of Cortisol and Lactate Using Graphene Embedded Screen-Printed Electrode. Nano-Micro Lett..

[91] Knieling T., Nebling E., Blohm L., Beale C., Fahland M. (2017). Printed and Flexible Electrochemical Lactate Sensors for Wearable Applications. Proceedings.

[92] Shi W., Luo X., Cui Y. (2018). A Tube-Integrated Painted Biosensor for Glucose and Lactate. Sensors.

[93] Zaryanov N.V., Nikitina V.N., Karpova E.V., Karyakina E.E., Karyakin A.A. (2017). Nonenzymatic Sensor for Lactate Detection in Human Sweat. Anal. Chem..

[94] Saha T., Songkakul T., Knisely C.T., Yokus M.A., Daniele M.A., Dickey M.D., Bozkurt A., Velev O.D. (2022). Wireless wearable electrochemical sensing platform with zero-power osmotic sweat extraction for continuous lactate monitoring. ACS Sens..

[95] Imani S., Bandodkar A.J., Mohan A.M.V., Kumar R., Yu S., Wang J., Mercier P.P. (2016). A Wearable Chemical–Electrophysiological Hybrid Biosensing System for Real-Time Health and Fitness Monitoring. Nat. Commun..

[96] Saha T., Del Caño R., Mahato K., De la Paz E., Chen C., Ding S., Wang J. (2023). Wearable electrochemical glucose sensors in diabetes management: A comprehensive review. Chem. Rev..

[97] Chen Q., Sun T., Song X., Ran Q., Yu C., Yang J., Feng H., Yu L., Wei D. (2017). Flexible Electrochemical Biosensors Based on Graphene Nanowalls for the Real-Time Measurement of Lactate. Nanotechnology.

[98] Chien M.-N., Fan S.-H., Huang C.-H., Wu C.-C., Huang J.-T. (2022). Continuous Lactate Monitoring System Based on Percutaneous Microneedle Array. Sensors.

[99] Ding Y., Yang L., Wen J., Ma Y., Dai G., Mo F., Wang J. (2025). A Comprehensive Review of Advanced Lactate Biosensor Materials, Methods, and Applications in Modern Healthcare. Sensors.

[100] Simic M., Manjakkal L., Zaraska K., Stojanovic G.M., Dahiya R. (2017). TiO_2_-Based Thick Film pH Sensor. IEEE Sens. J..

[101] Goh G.L., Agarwala S., Tan Y.J., Yeong W.Y. (2018). A Low Cost and Flexible Carbon Nanotube pH Sensor Fabricated Using Aerosol Jet Technology for Live Cell Applications. Sens. Actuators B Chem..

[102] Nyein H.Y.Y., Gao W., Shahpar Z., Emaminejad S., Challa S., Chen K., Fahad H.M., Tai L.-C., Ota H., Davis R.W. (2016). A Wearable Electrochemical Platform for Noninvasive Simultaneous Monitoring of Ca^2+^ and pH. ACS Nano.

[103] Oh S.Y., Hong S.Y., Jeong Y.R., Yun J., Park H., Jin S.W., Lee G., Oh J.H., Lee H., Lee S.-S. (2018). Skin-Attachable, Stretchable Electrochemical Sweat Sensor for Glucose and pH Detection. ACS Appl. Mater. Interfaces.

[104] Cao H., Landge V., Tata U., Seo Y.-S., Rao S., Tang S.-J., Tibbals H.F., Spechler S., Chiao J. (2012). An Implantable, Batteryless, and Wireless Capsule with Integrated Impedance and pH Sensors for Gastroesophageal Reflux Monitoring. IEEE Trans. Biomed. Eng..

[105] Guinovart T., Parrilla M., Crespo G.A., Rius F.X., Andrade F.J. (2013). Potentiometric Sensors Using Cotton Yarns, Carbon Nanotubes and Polymeric Membranes. Analyst.

[106] Qin Y., Kwon H.-J., Subrahmanyam A., Howlader M.M.R., Selvaganapathy P.R., Adronov A., Deen M.J. (2016). Inkjet-Printed Bifunctional Carbon Nanotubes for pH Sensing. Mater. Lett..

[107] Manjakkal L., Vilouras A., Dahiya R. (2018). Screen Printed Thick Film Reference Electrodes for Electrochemical Sensing. IEEE Sens. J..

[108] Pirovano P., Dorrian M., Shinde A., Donohoe A., Brady A.J., Moyna N.M., Wallace G., Diamond D., McCaul M. (2020). A wearable sensor for the detection of sodium and potassium in human sweat during exercise. Talanta.

[109] Jalal N.R., Madrakian T., Ahmadi M., Afkhami A., Khalili S., Bahrami M., Roshanaei M. (2024). Wireless wearable potentiometric sensor for simultaneous determination of pH, sodium and potassium in human sweat. Sci. Rep..

[110] De Matteis V., Cannavale A., Blasi L., Quarta A., Gigli G. (2016). Chromogenic device for cystic fibrosis precocious diagnosis: A ‘point-of-care’ tool for sweat testing. Sens. Actuators B Chem..

[111] Liao J., Zhang X., Sun Z., Chen H., Fu J., Si H., Ge C., Lin S. (2022). Laser-Induced Graphene-Based Wearable Epidermal Ion-Selective Sensors for Noninvasive Multiplexed Sweat Analysis. Biosensors.

[112] Li Y., Song S., Song J., Gong R., Abbas G. (2025). Electrochemical pH Sensor Incorporated Wearables for State-of-the-Art Wound Care. ACS Sens..

[113] Khan S., Ali S., Bermak A. (2019). Recent Developments in Printing Flexible and Wearable Sensing Electronics for Healthcare Applications. Sensors.

[114] Ahmad R., Tripathy N., Park J.-H., Hahn Y.-B. (2015). A Comprehensive Biosensor Integrated with a ZnO Nanorod FET Array for Selective Detection of Glucose, Cholesterol and Urea. Chem. Commun..

[115] Gahlaut A., Hooda V., Dhull V., Hooda V. (2018). Recent Approaches to Ameliorate Selectivity and Sensitivity of Enzyme Based Cholesterol Biosensors: A Review. Artif. Cells Nanomed. Biotechnol..

[116] Rajan A., Vishnu J., Shankar B. (2024). Tear-based ocular wearable biosensors for human health monitoring. Biosensors.

[117] Benjamin E.M. (2002). Self-Monitoring of Blood Glucose: The Basics. Clin. Diabetes.

[118] Freckmann G., Pleus S., Grady M., Setford S., Levy B. (2019). Measures of Accuracy for Continuous Glucose Monitoring and Blood Glucose Monitoring Devices. J. Diabetes Sci. Technol..

[119] Tang L., Chang S.J., Chen C.-J., Liu J.-T. (2020). Non-Invasive Blood Glucose Monitoring Technology: A Review. Sensors.

[120] Yu A., Zhu M., Chen C., Li Y., Cui H., Liu S., Zhao Q. (2024). Implantable flexible sensors for health monitoring. Adv. Healthc. Mater..

[121] Pantelopoulos A., Bourbakis N.G. (2010). A Survey on Wearable Sensor-Based Systems for Health Monitoring and Prognosis. IEEE Trans. Syst. Man Cybern. Part C Appl. Rev..

[122] Phipps J., Passage B., Sel K., Martinez J., Saadat M., Koker T., Damaso N., Davis S., Palmer J., Claypool K. (2024). Early adverse physiological event detection using commercial wearables: Challenges and opportunities. npj Digit. Med..

[123] Perez M.V., Mahaffey K.W., Hedlin H., Rumsfeld J.S., Garcia A., Ferris T., Balasubramanian V., Russo A.M., Rajmane A., Cheung L. (2019). Large-Scale Assessment of a Smartwatch to Identify Atrial Fibrillation. N. Engl. J. Med..

[124] Seshadri D.R., Davies E.V., Harlow E.R., Hsu J.J., Knighton S.C., Walker T.A., Voos J.E., Drummond C.K. (2020). Wearable Sensors for COVID-19: A Call to Action to Harness Our Digital Infrastructure for Remote Patient Monitoring and Virtual Assessments. Front. Digit. Health.

[125] Heikenfeld J., Jajack A., Rogers J., Gutruf P., Tian L., Pan T., Li R., Khine M., Kim J., Wang J. (2018). Wearable Sensors: Modalities, Challenges, and Prospects. Lab Chip.

[126] Zhang J., Mihai C., Tüshaus L., Scebba G., Distler O., Karlen W. (2021). Wound Image Quality from a Mobile Health Tool for Home-Based Chronic Wound Management with Real-Time Quality Feedback: Randomized Feasibility Study. JMIR mHealth uHealth.

[127] Jacobsen M., Gholamipoor R., Dembek T.A., Rottmann P., Verket M., Brandts J., Jäger P., Baermann B.-N., Kondakci M., Heinemann L. (2023). Wearable Based Monitoring and Self-Supervised Contrastive Learning Detect Clinical Complications during Treatment of Hematologic Malignancies. npj Digit. Med..

[128] Smuck M., Odonkor C.A., Wilt J.K., Schmidt N., Swiernik M.A. (2021). The Emerging Clinical Role of Wearables: Factors for Successful Implementation in Healthcare. npj Digit. Med..

[129] Dorsey E.R., Topol E.J. (2020). Telemedicine 2020 and the next Decade. Lancet.

[130] Badawy S.M., Cronin R.M., Hankins J., Crosby L., DeBaun M., Thompson A.A., Shah N. (2018). Patient-Centered eHealth Interventions for Children, Adolescents, and Adults with Sickle Cell Disease: Systematic Review. J. Med. Internet Res..

[131] Mendt S., Zout G., Rabuffetti M., Gunga H.C., Bunker A., Barteit S., Maggioni M.A. (2025). Laboratory comparison of consumer-grade and research-established wearables for monitoring heart rate, body temperature, and physical acitivity in sub-Saharan Africa. Front. Physiol..

[132] Banaee H., Ahmed M., Loutfi A. (2013). Data Mining for Wearable Sensors in Health Monitoring Systems: A Review of Recent Trends and Challenges. Sensors.

[133] Akhmetov A., Latif Z., Tyler B., Yazici A. (2025). Enhancing healthcare data privacy and interoperability with federated learning. PeerJ Comput. Sci..

[134] Büchter R.B., Betsch C., Ehrlich M., Fechtelpeter D., Grouven U., Keller S., Meuer R., Rossmann C., Waltering A. (2019). Communicating Uncertainty from Limitations in Quality of Evidence to the Public in Written Health Information: Protocol for a Web-Based Randomized Controlled Trial. JMIR Res. Protoc..

[135] Nissenbaum H. (2010). Privacy in Context: Technology, Policy, and the Integrity of Social Life.

[136] De Arriba-Pérez F., Caeiro-Rodríguez M., Santos-Gago J. (2016). Collection and Processing of Data from Wrist Wearable Devices in Heterogeneous and Multiple-User Scenarios. Sensors.

[137] Eng D., Chute C., Khandwala N., Rajpurkar P., Long J., Shleifer S., Khalaf M.H., Sandhu A.T., Rodriguez F., Maron D.J. (2021). Automated Coronary Calcium Scoring Using Deep Learning with Multicenter External Validation. npj Digit. Med..

[138] Mardanshahi A., Sreekumar A., Yang X., Barman S.K., Chronopoulos D. (2025). Sensing techniques for structural health monitoring: A state-of-the-art review on performance criteria and new-generation technologies. Sensors.

[139] Topol E.J. (2013). The Creative Destruction of Medicine: How the Digital Revolution Will Create Better Health Care.

[140] Bates D.W., Singh H. (2018). Two Decades Since *To Err Is Human*: An Assessment of Progress and Emerging Priorities in Patient Safety. Health Aff..

